# Antidepressant-Resistant Depression and Antidepressant-Associated Suicidal Behaviour: The Role of Underlying Bipolarity

**DOI:** 10.1155/2011/906462

**Published:** 2011-04-03

**Authors:** Zoltan Rihmer, Xenia Gonda

**Affiliations:** Department of Clinical and Theoretical Mental Health, Kútvölgyi Clinical Center, Semmelweis University, Kútvölgyi út 4., 1125 Budapest, Hungary

## Abstract

The complex relationship between the use of antidepressants and suicidal behaviour is one of the hottest topics of our contemporary psychiatry. Based on the literature, this paper summarizes the author's view on antidepressant-resistant depression and antidepressant-associated suicidal behaviour. Antidepressant-resistance, antidepressant-induced worsening of depression, antidepressant-associated (hypo)manic switches, mixed depressive episode, and antidepressant-associated suicidality among depressed patients are relatively most frequent in bipolar/bipolar spectrum depression and in children and adolescents. As early age at onset of major depressive episode and mixed depression are powerful clinical markers of bipolarity and the manic component of bipolar disorder (and possible its biological background) shows a declining tendency with age antidepressant-resistance/worsening, antidepressant-induced (hypo)manic switches and “suicide-inducing” potential of antidepressants seem to be related to the underlying bipolarity.

## 1. Introduction

Treatment-resistant and particularly antidepressant-resistant major depression (AD-RD) is a great clinical challenge both in the cases of unipolar and bipolar depression [1, 2]. While it is well documented that the optimal clinical response to antidepressants is much rare in bipolar I and II than in unipolar major depression [3–5] only the most recent clinical studies have focused on the boundaries between treatment-resistant unipolar major depressive disorder and bipolar disorder. These studies seem to be more promising in understanding both antidepressant-resistance and antidepressant-associated suicidal behaviour in patients with major mood disorders.

## 2. Antidepressant Resistance in Major Depressive Episode: Its Relationship with Bipolar Disorder

 The generally accepted definition of AD-RD refers that the depressed patient does not show a clinically significant response after at least two adequate trials of different classes of antidepressants. In spite of the fact that there are several causes of AD-RD in general [1, 6], one of the most common sources of it is the unrecognized bipolar nature of the “unipolar” major depressive disorder, when the patients receive antidepressant monotherapy—unprotected by mood stabilizers/atypical antipsychotics [4–11]. Unrecognized bipolar depressives are generally treated as “unipolar” major depressives which means that these patients do not receive mood stabilizers [3, 12]. This can result in a very high rate of treatment resistance, which is about two-times higher than in patients with true unipolar major depression [4–10]. The frequency of AD-RD ranges from 41% to 65% in bipolar I and II depression and between 18%–27% in unipolar depression [4–6, 10]. The rate of the bipolar spectrum disorder among the DSM-IV defined antidepressant responsive unipolar major depressive disorder inpatients was 3.8%, but the same figure in antidepressant-resistant inpatients was 47.1% [6] indicating that the underlying bipolar diathesis was important contributor to antidepressant nonresponse.

Antidepressant monotherapy in bipolar and bipolar spectrum depressives can worsen the cross-sectional picture of depression not only by resulting in (hypo)manic switch, but also via inducing or aggravating depressive mixed state/agitation, that is the major substrate of suicidal behaviour [7, 13–15]. The retrospective chart-review of 17 patients with “prebipolar” major depression (i.e., patients who become bipolar I and II during the followup) and of 17 pure unipolar depression showed that early onset of major depressive episode as well as treatment-emergent mixed depression, mood lability, psychomotor activation, suicidality, and nonresponse to antidepressant monotherapy were significantly more frequent in “prebipolar” than in pure unipolar depressives [10]. As early-onset major depression is a risk factor for bipolar depression [16–18] the higher frequency of antidepressant-induced mania [19] and the much lower rate of antidepressant response in children and adolescents than in adults [20] are also the reflexions of the bipolar nature of depression in these cases. A study on antidepressant-associated chronic irritable dysphoria (ACID) showed that it was significantly more common among bipolar I and II depressives who received antidepressants than among those who did not receive antidepressants, and the development of ACID (i.e., worsening of depression) was significantly related to past history of antidepressant-induced mood switches [14]. It is also known that bipolar spectrum and bipolar II depressives have frequently a loss of response to repeated trials of antidepressants (also called as tachyphyaxis) before developing chronic and severe AD-RD [8, 21]. 

The cross-sectional clinical picture of ACID [9, 14] is almost identical, with the clinical presentation of the depressive mixed state (= 3 or more intradepressive noneuphoric hypomanic symptoms) which is present in about 50%–60% of bipolar II depressives and around 20%–30% of unipolar depressives [22–27]. Family history data, demographic variables as well as long-term course, indicate that depressive mixed state, even in the frame of unipolar depression belongs to the broader bipolar spectrum [22–25, 28–30]. The recognition of depressive mixed state has important practical implications as several studies have shown that compared to pure major depressive episode the risk of suicidal behaviour is markedly elevated in mixed depressive episode both in DSM-IV diagnosed unipolar and bipolar depression [13, 22, 27, 31–34].

DSM-IV-defined unipolar major depressive disorder patients with subthreshold bipolarity convert more often into DSM-IV bipolar disorders than those with pure (unipolar) major depressive disorder [12], and maniform mood switches during antidepressant pharmacotherapy are significantly more common in bipolar than in unipolar depression. In addition, agitated/mixed major depression (even minimal hypomanic symptoms coexisting with otherwise full syndromal major depression) significantly predicts maniform switches during antidepressant treatment in patients with bipolar I and II depression [4, 6, 29, 35]. Because antidepressant-induced mood switch can be markedly reduced by coadministration of lithium or other mood stabilizers [4, 36–39] and antidepressant-induced worsening of depression (i.e., ACID) is significantly related to prior mood switches [14] it is very likely that co-administration of lithium/other mood stabilizers with antidepressants can reduce the risk not only of the (hypo)manic switch, but also of drug-resistance as well as developing AD-RD or worsening of depression in manifest or covert cases of bipolar depression. The well-documented positive effect of lithium (and atypical antipsychotic) augmentation in antidepressant-resistant unipolar major depressive disorder [1] and the marked antisuicidal effect of lithium in bipolar and unipolar mood disorder patients [40] also supports this postulation. The high rate of responders and remitters in patients receiving quetiapine monotherapy both in DSM-IV unipolar and bipolar depression [41, 42] also suggests that the antidepressant-resistance of these patients might be causally related, at least in part, to the underlying (hypo)manic component of the depressive episode.

## 3. Antidepressants and Suicidal Behaviour: The Role of Underlying Bipolarity

This phenomenon of AD-RD and antidepressant-induced mood switch is also related to “antidepressant-induced suicidal behaviour.” As suicidality decreases/vanishes after clinical recovery of depression, nonresponse to antidepressants or worsening of depression due to antidepressants or spontaneous worsening is an important suicide risk factor [33, 48]. The meta-analysis of the efficacy of SSRIs in children and adolescents with major depressive disorder showed that the rate of responders was significantly higher in patients receiving fluoxetine and citalopram/escitalopram than those receiving placebo, but there was no significant drug-placebo difference for patients receiving paroxetine and sertraline. In line with this, the relative risk of “antidepressant-associated” suicidal behaviour (attempts and ideations only) was much lower in fluoxetine and citalopram/escitalopram patients than in paroxetine and sertraline groups [43]. The rarely occurring antidepressant-associated suicidality among depressed patients is relatively most frequent in children and adolescents and less common in middle-aged persons and in the elderly both in RCTs [44–46] and observational studies [47]. As early age at onset of major depressive episode is a powerful clinical marker of bipolarity [16–18], and the manic component of bipolar disorder (and possible its biological background) shows a declining tendency with age [30]. The “suicide-inducing” potential of antidepressants, occurring almost exclusively among young patients, seems to be really related to the underlying bipolarity. This is supported by the finding that mixed major depressive episode—a well-considered suicide risk factor—is much more frequent in children and adolescents than in adults [49]. As combination of antidepressants and mood stabilizers reduces the “iatrogenic” hypomanic/manic mood switch [4, 29, 37–39] this treatment strategy could be also useful in avoiding both AD-RD and “antidepressant-associated” suicidal behaviour. This is important, because a significant part (30–40%) of DSM-IV diagnosed unipolar depressives really have subthreshold bipolar II or bipolar spectrum disorder [3, 12, 24, 25, 30] that means that about one-third of DSM-IV diagnosed unipolar depressives are really bipolar depressives. 

As antidepressants can worsen depression but it is not the case for placebo, this new view can explain why antidepressant-induced suicidal behaviour is more common among patients treated with antidepressants compared to those who receive placebo in Randomized Controlled Trials (RCTs) on antidepressant monotherapy in unipolar major depression: subthreshold bipolar depressives and bipolar spectrum depressives (including agitated depressives and depressed patients with family history of bipolar disorder) are not excluded from this trials resulting in a significant part of bipolar depressives among this study population [12, 30]. 

If the depression worsening and suicidal behaviour-inducing potential of antidepressants are largely limited for depressive patients only, particularly for those with overt or covert bipolar depression, no worsening of general condition or no depression-provoking effect and no increased suicidality in patients receiving antidepressants for other than depression indication would be expected. Indeed, the meta-analysis of 372 placebo-controlled randomised antidepressant trials showed that, for participants with nonpsychiatric indications (*N* = 22,024), suicidal behaviour is extremely rare and consisted almost entirely of suicidal ideation alone [46]. A most recent analysis of almost 15,000 patients from 57 placebo-controlled paroxetine trials on major depressive disorder and anxiety disorder showed no differences between paroxetine and placebo for suicidal behaviour. However, in the separate analysis of patients with major depressive disorder, the incidence of suicidal behaviour was significantly greater for paroxetine than for placebo (0.32% versus 0.05%, OR = 6.7) particularly among young adults aged 30 years or less. On the other hand, in the nondepression clinical trial database (*N* = 8931) the rates of suicidal behaviour in the paroxetine group and in the placebo group were almost identical (0.13% and 0.11%, resp.) [45]. 

Worsening of medical state, in which the given drugtherapy is effective in general, but could at times be problematic, is also a problem in other fields of medicine. Provocation of a new arrhythmia or increase in the frequency of preexisting arrhythmia occurs with all antiarrhythmic drugs in 6%–23% of cases, particularly among those patients with underlying heart disease and evidence of ongoing overt or salient cardiac ischemia. As a rule, aggravation of arrhythmia occurs mainly in the first few days of initiating therapy but occurrence of a new arrhythmia can also be a late complication of it [50, 51].

## 4. Conclusions

There is no doubt that successful acute and long-term antidepressant pharmacotherapy of depressive patients significantly reduces the risk of suicidality in the vast majority of patients but antidepressant monotherapy (unprotected by mood stabilizers or atypical antipsychotics) is frequently ineffective and rarely can worsen bipolar depression and therefore increase the suicide risk in a small, vulnerable subpopulation of them. Antidepressants cannot “cause” suicidal behaviour but, as discussed above, in contrary to placebo, they can worsen depression. As suicidality in depressed patients is also severity-dependent phenomenon worsening of depression is the main final “cause” of suicidal behaviour even in drug-free depressives. When antidepressant monotherapy worsens depression (and consequently increases the suicide risk) in relatively few patients, its psychopathological substrate might well reside in an agitated, excited, mentally overstimulated depressive mixed state that arises primarily from the underlying bipolar diathesis (see Table 1). The pattern the clinical response to antidepressant monotherapy in major depressive episode seems to be lied is on a continuum, the different steps of which are (1) optimal response (response/remission), (2) no significant change, (3) worsening of depression (covert switch = occurrence of intradepressive hypomanic symptoms = antidepressant-induced depressive mixed state), and (4) hypomania or mania (overt switch). Psychiatrists should identify persons who might be not helped or threatened by a given intervention. The formal recognition of subthreshold bipolar (bipolar spectrum) disorders, particularly of depressive mixed states, in our official diagnostic systems will help to identify those depressives who do not respond/worsen to antidepressant monotherapy and consequently they are at increased risk of suicidal behaviour [15, 22]. Incorporating these new findings into everyday clinical practice is urgently needed. 

## Figures and Tables

**Table 1:** The role of underlying bipolarity in antidepressant-resistance and antidepressant-associated suicidality in patients with mood disorders.

The frequency of antidepressant-resistance is about double in bipolar (I + II) and bipolar spectrum disorder than in unipolar depression [4–6, 10]	

Antidepressant monotherapy can worsen the cross-sectional picture and long-term course of bipolar (I + II) and bipolar spectrum disorder [7, 9, 10, 13–15]	

Early-onset major depressive episode is (pre)bipolar [16–18, 30]	

Children and adolescents with major depressive episode are less responsive to antidepressants than adults [20, 43]	The rarely occurring antidepressant-associated suicidal behavior among depressed patients is relatively most frequent in children and adolescents [43–47]
